# Elevated and Sustained Intracellular Calcium Signalling Is Necessary for Efficacious Induction of the Human Sperm Acrosome Reaction

**DOI:** 10.3390/ijms231911253

**Published:** 2022-09-24

**Authors:** Priyanka Prajapati, Shruti Kane, Rachel C. McBrinn, Morven S. Dean, Sarah J. Martins da Silva, Sean G. Brown

**Affiliations:** 1 Reproductive Medicine Research Group, School of Medicine, Ninewells Hospital and Medical School, University of Dundee, Dundee DD1 9SY, UK; 2 School of Applied Sciences, Abertay University, Dundee DD1 1HG, UK; 3 Assisted Conception Unit, Ninewells Hospital, Dundee DD1 9SY, UK

**Keywords:** sperm physiology, acrosome reaction, human sperm, CatSper, ion channel, progesterone, prostaglandins, intracellular calcium, fertilisation

## Abstract

Progesterone and prostaglandin E1 are postulated to trigger the human sperm acrosome reaction (AR). However, their reported efficacy is very variable which likely, in part, reflects the plethora of experimental conditions and methodologies used to detect this physiologically relevant event. The purpose of this study was to develop an assay for the robust induction and objective measurement of the complete AR. Sperm from healthy volunteers or patients undertaking IVF were treated with a variety of ligands (progesterone, prostaglandin E1 or NH_4_Cl, alone or in combinations). AR, motility and intracellular calcium measurements were measured using flow cytometry, computer-assisted sperm analysis (CASA) and fluorimetry, respectively. The AR was significantly increased by the simultaneous application of progesterone, prostaglandin E1 and NH_4_Cl, following an elevated and sustained intracellular calcium concentration. However, we observed notable inter- and intra-donor sample heterogeneity of the AR induction. When studying the patient samples, we found no relationship between the IVF fertilization rate and the AR. We conclude that progesterone and prostaglandin E1 alone do not significantly increase the percentage of live acrosome-reacted sperm. This assay has utility for drug discovery and sperm toxicology studies but is not predictive for IVF success.

## 1. Introduction

The acrosome reaction (AR) is a profound phenotypical event observed in capacitated human sperm and involves the fusion of the outer acrosome membrane with the plasma membrane and the release of the acrosomal contents [1]. This Ca^2+^-dependent process is a prerequisite for sperm fertilisation competence, as it results in the exposure of the proteins on the inner acrosome membrane required for oocyte plasma membrane-binding and fusion [2]. In several mammals, it has been observed that intact and solubilised zona pellucida (ZP) trigger the AR [3,4,5,6,7,8,9]. However, innovative studies using genetically modified mouse models have shown that the AR is triggered in most sperm remote from the ZP surface [10,11,12]. The sequence of events in humans in vivo is not known, but some sperm are observed to begin the AR as they penetrate the cumulus layers during IVF [13], and the cumulus cell-conditioned media triggers the AR [14], indicating the involvement of a soluble inducer(s). Progesterone (P4) is produced by cumulus cells [14,15] and is proposed to mediate the follicular fluid-induced increase in the intracellular calcium, [Ca^2+^]i, [16,17,18] and the AR [19,20].

P4 is a potent and efficacious agonist of [Ca^2+^]i through the indirect activation of the sperm-specific calcium ion channel CatSper [21,22]. The activity of the CatSper is critical for sperm fertilisation in vivo and at IVF [23,24]. Impaired P4-induced AR has been reported in some patients [25,26] and, correspondingly, several studies have associated this with failed P4-induced [Ca^2+^]i response/CatSper function [24,27,28,29], indicating that AR measurement may be a useful phenotypical screen to assess P4-signalling [30] and a potential tool for the investigation of impaired fertilization at IVF. However, there are conflicting reports regarding the predictive value of P4-induced AR assays for IVF success [31,32], and there are considerable discrepancies between the studies regarding the efficacy of P4 at inducing the AR in sperm from healthy volunteers, with several studies even reporting that it is ineffective [33,34,35,36,37,38,39,40]. The inter- and intra-donor variability are likely to account for the discrepancies described [37,41,42,43], and the differences in the experimental methodologies are also likely to be important [44]. Nonetheless, understanding the reasons for the disparities between the studies is imperative to permit informative studies of sperm (patho)physiology. Therefore, in this study, we sought to revisit and standardise the experimental methodology and to develop a reliable and reproducible assay for the robust induction and measurement of the physiologically relevant complete AR in healthy human sperm. To our knowledge, this study is unique in directly comparing the effects of P4, prostaglandin E1 (PGE1) and NH_4_Cl on pH, Ca^2+^, motility and acrosome reaction.

## 2. Results

**Figure 1:**
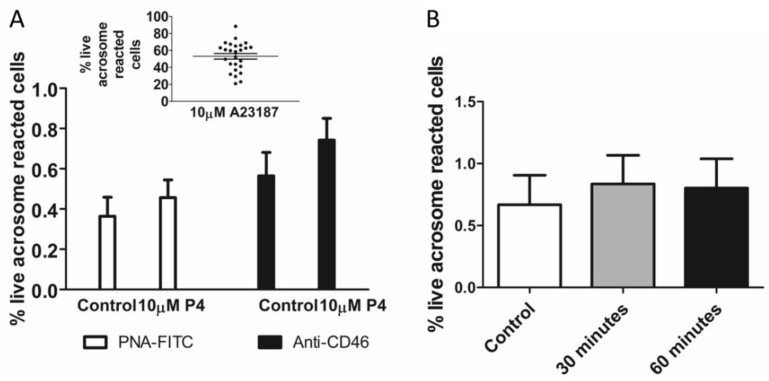
P4 does not significantly increase completed acrosome reaction (AR). (**A**). Measurement of the AR after 30-min exposure to P4 (10 μM) in density gradient prepared human sperm incubated in capacitating conditions for 3 h. There is no significant difference between the percentage of acrosome reaction reported by flow cytometry using PNA-FITC or anti-CD46 APC-conjugated monoclonal antibody of sperm incubated with 10 μM P4 for 30 min (paired data of 26 samples from 18 donors). AR induction by ionophore A23187 in these samples detected using anti-CD46 is shown in insert. (**B**). Incubation of sperm with P4 for 60 min does not significantly increase AR (*n* = 5 samples from 5 donors). Addition of vehicle (DMSO) served as a negative control.

We first compared the AR reported by lectin PNA-FITC and an anti-CD46 monoclonal antibody in spermatozoa prepared by density gradient centrifugation and incubated in capacitating conditions for three hours. The exposure of the cells to the P4 (10 μM) for 30 min did not significantly increase the AR (Figure 1A, *n* = 26 samples from 18 donors) although, in some cases, there was >2-fold increase. There was a highly variable AR induction by the positive control (calcium ionophore A23187, Figure 1A insert) in each case. There was no significant difference in the AR induction reported between the two markers. Due to the perceived benefits of using the anti-CD46 over the PNA-FITC (see discussion), we opted to use the anti-CD46 going forward.

Although the previous published studies (see Supplementary Table S1) of individual and cell populations indicate that P4 induces an increase in acrosome-reacted cells after 20–30 min, we demonstrated that P4 was ineffective, even when extending the incubation period to 60 min (Figure 1B).

**Figure 2:**
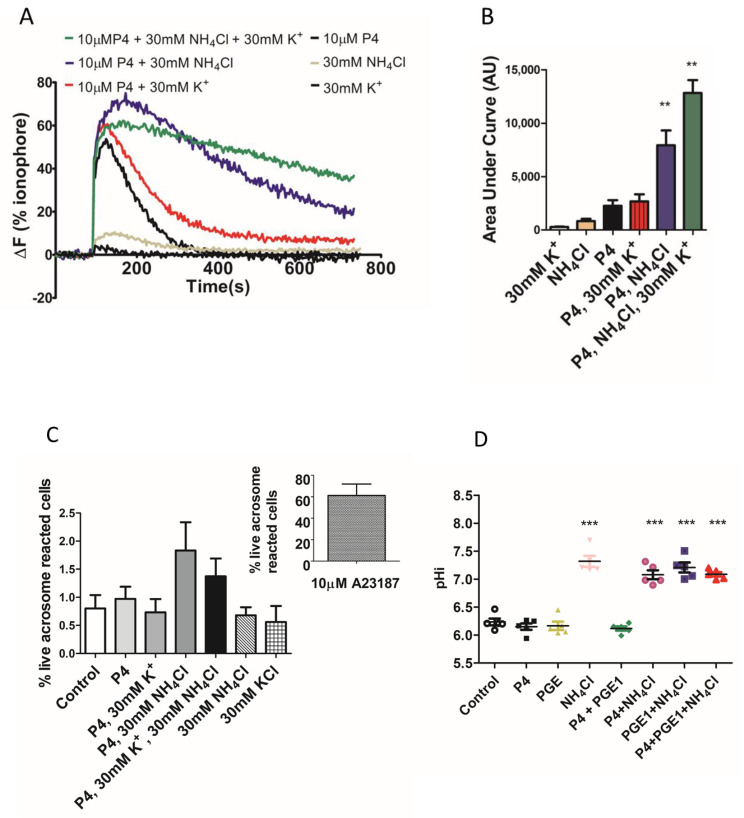
Co-application of NH_4_Cl and KCl significantly increases [Ca^2+^]i but not acrosome reaction. (**A**). Mean [Ca^2+^]i profiles induced by 30 mM K^+^, NH_4_Cl and P4 alone or in combination. (**B**). The total [Ca^2+^]i response to P4 is significantly increased by co-application of NH_4_Cl and NH_4_Cl/KCl to elevate extracellular K^+^ to 30 mM. AU = Arbitrary units. ** *p* < 0.01. (**B**). The acrosome reaction is not induced by any combination ((**C**), *n* = 5 from 5 donors). ΔF (%) is the percentage change in intensity (see methods) (**D**). NH_4_Cl (30 mM) alone and in combination with P4 (10 μM), PGE1 (5 μM) and P4/PGE1 significantly increases intracellular pH. Addition of vehicle (DMSO) served as a negative control. *** *p* < 0.001.

**Figure 3:**
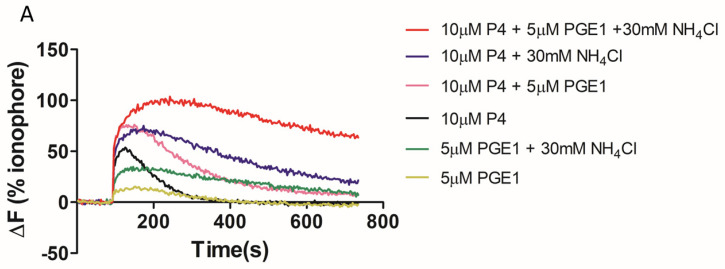

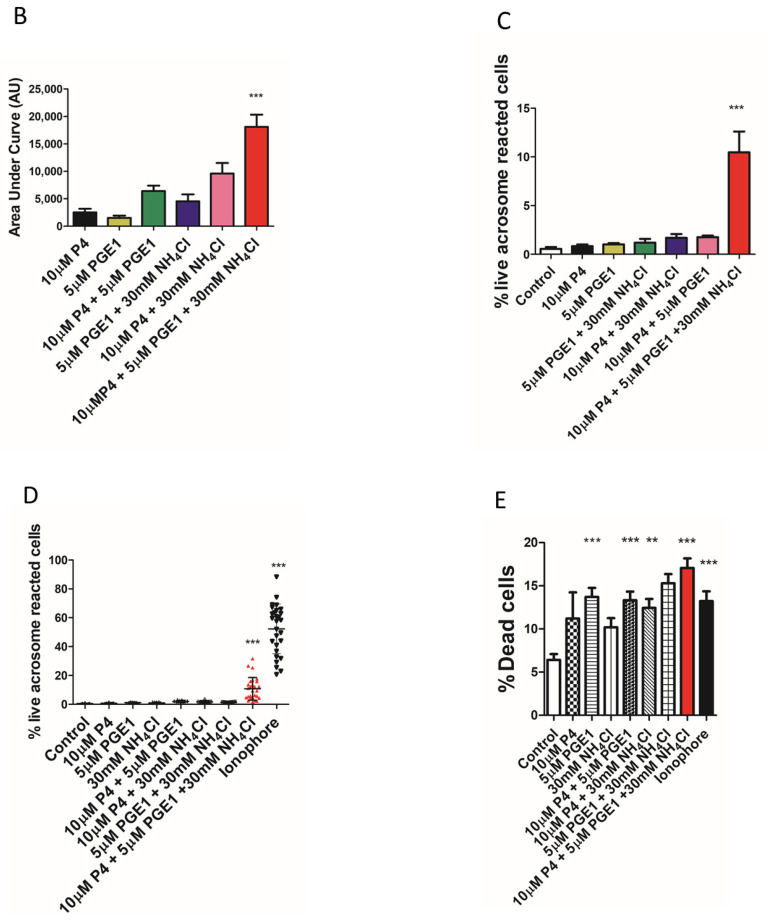
Co-application of progesterone (P4), prostaglandin E1 (PGE1) and NH_4_Cl significantly increases total intracellular calcium and acrosome reaction. (**A**). [Ca^2+^]i responses to 10 μM P4, 5 μM PGE1 alone and in combination with 30 mM NH_4_Cl. (**B**). Quantification of mean area under the curves in (**A**). The total [Ca^2+^]i response to P4/PGE1/NH_4_Cl is significantly greater than the response elicited by other combinations. AU = arbitrary units (**C**). Only P4/PGE1/NH_4_Cl significantly increases the acrosome reaction. Data for figures (**A**–**C**) are paired data using five samples from four donors. (**D**). The percentage of live cells that have completed the acrosome reaction in response to P4/PGE1/NH_4_Cl showed high inter-donor heterogeneity but was significantly different to all other conditions (*n* = 27 from 18 donors). (**E**). The percentage of dead cells under each experimental condition in (**D**). Data in figure (**D**,**E**) are paired and presented mean ± SD and are analysed using the Kruskal–Wallis test followed by Dunn’s post-test. Addition of vehicle (DMSO) served as a negative control. ^**^ and ^***^ represents statistical significance of *p* < 0.01 and <0.001 respectively.

**Figure 4:**
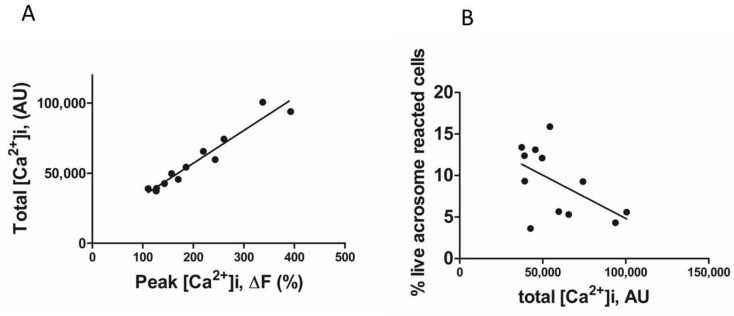
The relationship between P4/PGE1/NH_4_Cl-induced [Ca^2+^]i response and acrosome reaction induction. (**A**). There was a strong positive correlation (r2 = 0.94) between peak and total [Ca^2+^]i. AU = arbitrary units. (**B**). There was a weak negative correlation (r2 = −0.28) between total [Ca^2+^]i and percentage of cells that have completed the acrosome reaction. Data are paired from 12 samples from 11 donors.

**Figure 5:**
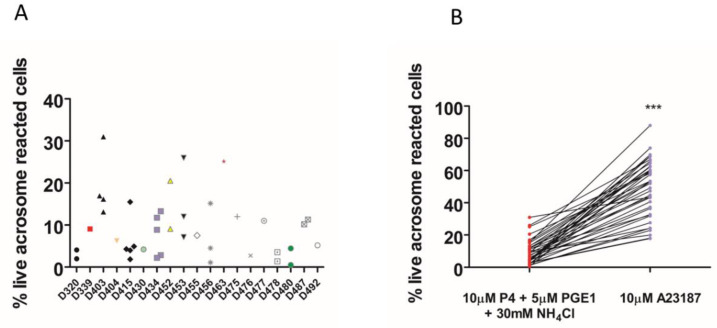
Intra-donor heterogeneity of acrosome reaction (AR) triggered by P4/PGE1/NH_4_Cl. (**A**). Scatter plot showing the variability of AR in 39 samples from 19 donors. (**B**). Paired data from the same experiments showing the response to ionophore relative to P4/PGE1/NH_4_Cl. Mean data are significantly different. *** *p* < 0.001.

**Figure 6:**
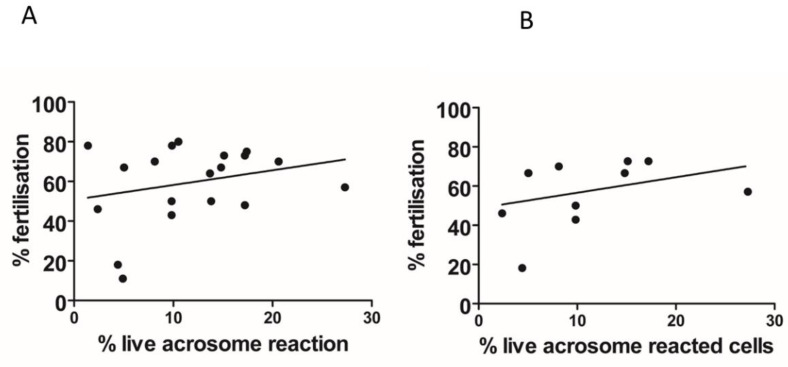
Relationship between the acrosome reaction triggered by P4/PGE1/NH_4_Cl and IVF success rate. (**A**). All patient data. (**B**). Couples with unexplained infertility.

**Figure 7:**
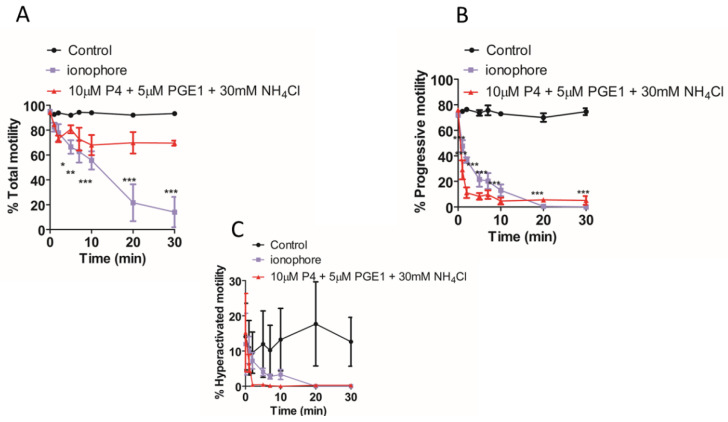
Simultaneous application of progesterone (P4), prostaglandin (PGE1) and NH_4_Cl impairs progressive and hyperactivated motility over 30 min exposure. (**A**). Total motility is significantly reduced (*p* < 0.05) by ionophore but not P4/PGE1/NH_4_Cl (PPN). PPN and ionophore significantly (*p* < 0.001) reduce progressive (**B**) and hyperactivated motility (**C**), *n* = 5. Data were analysed using repeated measures ANOVA followed by Tukey post-hoc test. Addition of vehicle (DMSO) served as a negative control. * *p* < 0.05; ** *p* < 0.01, *** *p* < 0.001.

We found that the total [Ca^2+^]i (see methods, [Ca^2+^]i measurement) was significantly higher when NH_4_Cl or NH_4_Cl/KCl was co-applied with the P4 (Figure 2A,B). However, in paired experiments, we found that no combination of the compounds significantly increased the AR (Figure 2C). The mean positive control data for all conditions in 2C are shown in the inset. Although NH_4_Cl significantly increases intracellular pH, we also demonstrated that P4 or PGE1 do not act synergistically with the NH_4_Cl to alter the intracellular pH (Figure 2D). We subsequently examined the [Ca^2+^]i responses to P4, PGE1 and NH_4_Cl alone or in combinations and, in parallel, measured the AR in cells from the same sample. As demonstrated previously, PGE1 is less efficacious than P4, even when co-applied with NH_4_Cl (Figure 3A). The co-application of PGE1, NH_4_Cl or PGE1/NH_4_Cl significantly increased the total [Ca^2+^]i compared to the P4 alone. The co-application of P4/PGE1/NH_4_Cl (PPN) significantly increased the total [Ca^2+^]i, compared to all other treatments (Figure 3B), and was the only condition that significantly increased the live cell AR (Figure 3C,D). Interestingly, most of the agonist combinations significantly increased cell death (Figure 3E). To explore the variability in the AR between the samples, we assessed the relationship between the [Ca^2+^]i response to the PPN and the AR in cells from the same donor sample. There was a strong positive correlation between the peak [Ca^2+^]i and total [Ca^2+^]i (r^2^ = 0.94, Figure 4A), but a weak negative relationship between the total [Ca^2+^]i and the percentage of live acrosome-reacted cells (Figure 4B). Collectively, these data indicate that the total [Ca^2+^]i is a critical, but not the sole, determinant for a robust AR induction by the physiological agonists under our experimental conditions. The biological variability is emphasized further by assessment of the inter- and intra-donor variability of the AR induced by the PPN (Figure 5A). It is of note that one sample, from D456, was relatively insensitive to the PPN (<2-fold change) and one sample, from D480, was insensitive to the PPN (1.2-fold change), despite an ionophore inducing a 20-fold increase in the AR in both cases. The general trend for the AR induction by an ionophore was that it was notably greater than that to the PPN for any given sample (Figure 5B). The PPN increased the AR an average of approximately 32-fold, compared to 1.6-fold for the P4 (paired data, see Supplementary Table S1). We found no relationship between the IVF fertilization rate and the AR induced by the PPN (Figure 6). The ionophore and PPN significantly reduced all the motility parameters (Figure 7A–C). The ionophore significantly reduced the total motility, compared to the PPN (Figure 7A). The majority of the PPN-treated spermatozoa were observed to be non-progressively motile (see the Supplementary Videos S1–S4).

**Figure 8:**
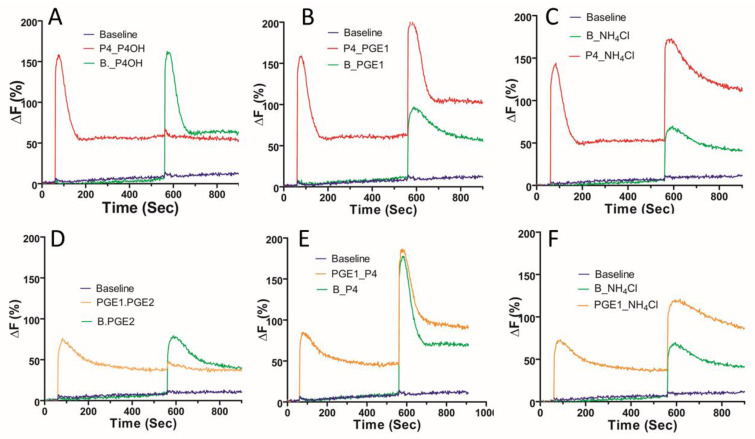
Examination of agonist cross-desensitization. Population mean [Ca^2+^]i traces (*n* = 5) showing initial agonist addition of either a saturating concentration of 10 μM progesterone (P4) (**A**–**C**) or 5 μM prostaglandin E1 (PGE1) (**D**–**F**), followed by the second agonist addition (17-OH progesterone (P4OH); prostaglandin E2 (PGE2)). A baseline control (shown in blue) was included in each experiment (sEBBS, represented as “B”). Addition of time control for the second agonist is shown in green. Cross-desensitisation experiments are shown in red (top panels) and orange (bottom panels). ΔF (%) is the percentage change in intensity (see methods).

**Figure 9:**
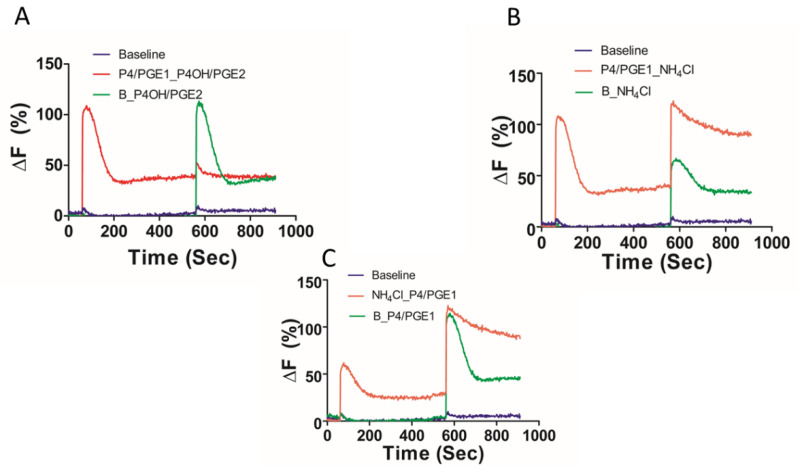
Asynchronous application of NH_4_Cl can sustain the [Ca^2+^]i response to progesterone and prostaglandin E1. Population mean [Ca^2+^]i traces (*n* = 5) showing initial agonist addition of either a saturating concentration of 10 μM progesterone (P4) or 5 μM PGE1 (**A**,**B**) or 30 mM NH_4_Cl (**C**) followed by a second agonist challenge. Baseline control (HTF, shown in blue) was included in each experiment. Addition of time control for the second agonist challenge is shown in green. A. Cross-desensitization of P4 and PGE1 responses. (**B**). Reactivation of CatSper following desensitization is achieved by additional of NH_4_Cl in the presence of P4/PGE1. (**C**). An elevated and sustained [Ca^2+^]i response to P4/PGE1 is achieved with pre-application of NH_4_Cl.

Lastly, we conducted cross-desensitization experiments to explore the mode of action of the various CatSper agonists, including the elevation of the intracellular pH, on the [Ca^2+^]i response. Pretreatment with P4 caused a desensitisation of the response to 17-OH-progesterone (P4OH) but not to PGE1 (Figure 8A). The response to the prostaglandin E2 (PGE2) was prevented by pretreatment with PGE1 (Figure 8D). In contrast, the responses to P4 and PGE1 did not cross-desensitize (Figure 8B,E). The application of NH_4_Cl following P4 or PGE1 caused a robust potentiation of the [Ca^2+^]i response (Figure 8C,F), with the peak [Ca^2+^]i response to NH_4_Cl being significantly greater in the presence of P4 (*p* = 0.0017) than the paired response to NH_4_Cl only. Remarkably, NH_4_Cl rescued the [Ca^2+^]i response, following desensitisation of the P4/PGE1 response (Figure 9B). The slowly decaying profile of the [Ca^2+^]i response was observed, even when NH_4_Cl was applied 5 min before the co-application of P4/PGE1 (Figure 9C).

## 3. Discussion

The P4-induced acrosomal exocytosis in capacitated human sperm (the acrosome reaction) is postulated to be a phenotypic reflection of the CatSper activation [24,36]. However, despite the efficacy of the P4 to increase the [Ca^2+^]i (~98% cells are responsive [30], its efficacy at inducing the AR in donor sperm is low and has been reported to be ineffective [33,45]. In this study, we aimed to develop a reliable and reproducible assay for a robust induction and measurement of the complete AR in human sperm using physiologically relevant agonists. We determined that a complete exocytosis is significantly enhanced only by the elevated and sustained [Ca^2+^]i achieved by the simultaneous challenge with P4, PGE1 and intracellular alkalinization. However, the percentage of cells undergoing the AR is highly variable and does not correlate to the [Ca^2+^]i response or fertilisation rate at IVF.

There are numerous published AR measurement protocol variations, including differences in the methods of isolating sperm from semen (swim-up or density gradient), media formulations (ion and energy substrate concentrations, type and concentration of serum), incubation time in capacitating conditions (3–24 h), P4 concentration (3–1590 mM) and exposure times (5 min to 24 h), AR indicator (lectins, membrane dyes or antibodies to inner acrosomal membrane markers), method of detection (manual, live cell imaging or flow cytometry) and inconsistent assessment of viability, which may contribute to the discrepancy in the spontaneous and P4-induced AR (see Supplementary Table S2 for a summary of selected publications). A systematic testing of all permutations is not feasible, so we focused on developing a robust methodology to measure the physiologically relevant complete acrosomal exocytosis. A manual assessment is limited by the slow, subjective nature of the scoring of fixed cells and, while single-cell imaging has been used to measure the kinetics of the AR in real-time [46,47], this method is not suitable for routine measurement, given its specialist and laborious nature. In contrast, flow cytometry permits the rapid, automated and standardized measurement of the acrosomal status of thousands of live cells.

Despite their routine use, the use of lectin dyes is potentially problematic. PSA has been shown to have a high background signal, bind acrosomal contents and report partial and complete AR [41], but the sperm that have completed the AR have diminished acrosomal labelling [48]. PNA gives a heterogenous acrosome-staining pattern [49], which is likely due to its binding to the outer acrosomal membrane and acrosomal vesicles [50] and so may also report an incomplete AR. In contrast, a monoclonal anti-CD46 antibody (clone E4.3) is specific for a marker of inner acrosome membrane exposure and may be a more representative marker of the completed AR [46]. Its use with an ionophore in flow cytometry has been suggested to be a sensitive and specific assay for the prediction of IVF success [51] and was therefore chosen as the marker for a complete acrosome reaction. Although we did not find any difference between the AR reported by the PNA and the anti-CD46 antibody, we determined that the P4 did not significantly increase the AR, even after a 1 h exposure. This is not a unique observation, but single-cell imaging data suggest that approximately 10% of sperm (5-fold greater than occurred spontaneously) complete the AR within 25 min following a P4 challenge [46]. The previous studies suggest that P4 increases the percentage of reacted sperm by approximately 2-5-fold [35,46], which we observed in some samples (Supplementary Table S1). Nevertheless, the measured absolute acrosome-reacted cell numbers are low, and the mean data are not significant. Therefore, we conclude that P4 is not an efficacious agonist for use for the induction of complete acrosomal exocytosis, but that it does trigger a complete exocytosis in a low percentage of cells in some samples.

Given the [Ca^2+^]i-dependent nature of the AR, we explored physiologically relevant strategies to increase the [Ca^2+^]i viaCatSper, which is sensitive to intracellular alkalinization, membrane potential depolarization and several prostaglandins [21,22]. The uterine tubule extracellular concentration of K^+^ is reported to be up to approximately 30 mM [52] and the elevated extracellular K^+^ increases the [Ca^2+^]i [53,54] and the AR [53,55]. However, the AR was not significantly increased in the cells incubated with P4 in 30 mM extracellular K^+^ or elevated intracellular pH conditions, despite the alkalinization acting with the P4 to sustain significantly larger total [Ca^2+^]i levels. The P4 and PGE1 synergistically activateCatSper [56] to potentiate the peak [Ca^2+^]i response, but only the simultaneous alkalinization caused a sufficiently large, sustained response to significantly increase the AR.

CatSper is a highly complex multimeric channel that is proposed to be negatively regulated by the endogenous endocannabinoid 2-arachidonoylglycerol, which is metabolized by the P4-sensitive lipid hydrolase α/β hydrolase domain-containing protein 2 [57]. In contrast, PGE1 activates CatSper via an alternative, unknown mechanism. In mouse sperm, the Ca^2+^ entry is limited by the coordinated actions of the CatSper subunits EFCAB9 and CatSperz, and the intracellular alkalinization causes EFCAB9 to dissociate from CatSperz to permit the maximum Ca^2+^ entry [58]. Important future work is to determine if a similar mechanism operates in human sperm, and the process by which the P4 and prostaglandins facilitate it.

The physiological regulation of the pHi in human sperm may involve bicarbonate ion transport, Na^+^/H^+^ exchange and/or a proton channel. The elevation of the extracellular bicarbonate ions increases the pHi in non-capacitated sperm [59], which may occur due to the action of bicarbonate ion transporters, such as SCL4 and SCL26 [60]. Alternatively, a Na^+^ dependent, bicarbonate ion independent exchange may regulate the pHi and AR [61], which may involve the activity of a Na^+^/H^+^ exchanger expressed in the principal piece [62]. The Hv1 proton channel is uniquely expressed in the flagella of the human sperm, and electrophysiological data show that Vm-depolarization evokes outward proton currents that are augmented by albumin, capacitation, fatty acids (oleic/arachidonic acid) and the endocannabinoid arachidonylethanolamide (anandamide) and are blocked by zinc ions [63]. Functional studies have shown that capacitated sperm, treated with a stable analogue of anandamide, exhibit acrosomal modifications which may represent partial AR [64] and that the AR is inhibited by Zn^2+^ [65] and by a Hv1-specific toxin [66]. Collectively, these data suggest that Hv1 is involved in regulating the AR.

The data are ambiguous regarding the location and timing of the initiation and completion of the AR for the successful fertilizing spermatozoon. The studies of genetically modified mouse models, which produce sperm that express fluorescent acrosome markers to permit live imaging in/ex vivo [11,12,67], demonstrated that the AR is complete in approximately 60–70% of the sperm by the time they reach the upper isthmus [11], and the acrosome-reacted sperm can penetrate and fertilize cumulus-intact oocytes [11,67]. These observations are in keeping with those from other animal studies [10,68,69] that suggest that the components (e.g., the cumulus oocyte complex) and/or ligand(s) produced and released around the time of ovulation at least initiate the AR before, or as, the sperm penetrate the cumulus mass. However, it has recently been demonstrated that solubilized zona pellucida (ZP) triggers a pHi and CatSper-dependent AR [70]. In humans, although induction of the AR using follicular fluid is not a universal observation [71], the weight of evidence is that the cumulus-oocyte complex, cumulus cells and developing follicles all produce ligand(s) that initiate, or at least prime [4,72], the sperm to undergo the AR. The CatSper agonists P4, prostaglandin A1, PGE1, PGE2 and prostaglandin F1a are present in human follicular fluid [73]; P4 is produced by the cumulus cells and the prostaglandin-endoperoxide synthase 2 (cyclooxygenase 2) transcripts are expressed in the cumulus cells [74], which suggests, as reported for the mouse [75], that human cumulus cells can synthesize and release prostaglandins. Additionally, there may be an increasing gradient of PGE/PGF production along the tubule and the PGE2 concentration increases in the ampullary and isthmic regions of the oviduct during the luteal phase [76]. The cumulus cell N-acyl phosphatidylethanolamine-specific phospholipase D (NAPE-PLD) gene expression, which regulates the anandamide synthesis, is elevated in women receiving human menopausal gonadotropin for controlled ovarian stimulation [77]. Thus, one may postulate that, as the sperm reach and penetrate the cumulus oophorous, they experience a combination of elevated pH [78] and ligands, which reach a sufficiently high local concentration to affect the ion channel function and trigger the AR. However, although elevated and sustained [Ca^2+^]i is necessary to trigger the AR, it is evident that, beyond an unknown threshold, it is counterproductive for motility (Figure 7, see also [79]).

We found no association between the PPN-mediated AR induction and IVF fertilisation rate, which is in keeping with the analysis of the studies examining the predictive potential of P4-mediated AR [44]. The acrosome-reacted and acrosome-intact human sperm bind the ZP [80,81]; the solubilized ZP increases in the [Ca^2+^]i [82,83,84] and the AR is triggered by the intact and solubilized ZP [5,82,85,86]. Therefore, as has been suggested for the mouse sperm [11,67], the nature of the trigger may not be of great significance but, rather, the fact that the human sperm possess the potential to react. Alternatively, the sperm that are triggered to the AR prior to contact with the ZP may be selected against or serve an alternative cooperative function, such as dispersing the hyaluronic matrix of the cumulus oophorous to facilitate the passage of the fertilizing spermatozoon.

The fold-change of the AR induced by the PPN was very variable, and we observed notable inter- and intra-donor heterogeneity (~1.2-242-fold change, Supplementary Table S1), the reasons for which are unknown. In vitro, the incubation of human sperm in capacitating conditions is associated with changes in the plasma membrane [87], protein phosphorylation [88], membrane potential (Vm) hyperpolarization [89] and elevation of the [Ca^2+^]i and pHi. All are necessary for cells to undergo the AR [47,90]. The AR is more likely to be suppressed in cells which exhibit Vm and pH-dependent [Ca^2+^]i oscillations [90,91], which occur spontaneously in ~35% of the capacitated cells [47], and ~20% develop oscillations in response to the P4 [30]. The Vm and pHi changes associated with capacitation are heterogeneous [89,92,93,94,95]. A spectrum of time-dependent membrane and tyrosine phosphorylation are documented to occur during the capacitation in human [96] and animal sperm [97,98,99] and P4 fails to trigger a [Ca^2+^]i response in approximately 2% of cells [30]. Thus, although these phenomena have been documented and in silico modelling attempted [92,100], the nature, cause and regulation of the molecular heterogeneity and consequence for the cellular function are poorly understood. For example, our data are in keeping with the principle of a [Ca^2+^]i threshold necessary to trigger function, as observed by the ionophore-induced hyperactivation in mouse sperm [79]. The immobilisation of mouse sperm has been reported following calcium ionophore exposure, with the flagellar activity ultimately relating to the [Ca^2+^] [79]. Whilst a reduction of the progressive and hyperactivated motility in response to the maximal CatSper ligand activation would appear to be counterintuitive in the context of the sperm function required for fertilisation, it is possible that our findings reflect the use of supra-physiological agonist concentrations in vitro. Therefore, the expression and regulation of the CatSper, the thresholds and mechanisms for reaching and interpreting the [Ca^2+^]i signal for the sperm function and the consequence of the molecular heterogeneity on the fertilisation competence are important and challenging future areas of study. Studying the many factors and permutations implicated in triggering and controlling the AR is made more challenging as they must be done at a single-cell level and at a scale to accurately capture the cellular physiological inter/intra donor variability. Understanding the variation is particularly important, given that the deficient P4-induced AR is associated with a defective CatSper expression/activity and male infertility [26,28,29,32,36]. Given the limited efficacy of the P4 and the potential dependence on the CatSper for ZP-induced exocytosis, there are still open questions regarding the nature of the phenotypic impairment in the CatSper-null human sperm that results in fertilisation failure.

In summary, we have demonstrated that the synergistic actions of P4 and PGE1 with the synchronous elevation of pHi significantly increase the total [Ca^2+^]i and significantly increase the complete acrosomal exocytosis in human sperm. It is important to determine the regulation and physiological relevance of this induction, particularly as we observe heterogeneity in the efficacy of the induction within and between the donors and find no association to the fertilisation rate in the patient sperm at IVF. The important next steps are to understand the regulatory interdependence of the sperm ion channels [101] and the influence of the capacitation. Finally, this method of induction will have great utility in drug discovery [102] and toxicology studies [39,40].

## 4. Materials and Methods

### 4.1. Chemicals, Reagents, and Antibodies

All the chemicals were purchased from Sigma-Aldrich, UK, unless stated otherwise. The sperm preparation gradients (PureCeption 40% and 80%) and Quinn’s Advantage Sperm Washing Medium (SWM) were purchased from CooperSurgical, Inc., US. The allophycocyanin (APC)-conjugated Mouse Anti-Human CD46 (Clone E4.3) was purchased from BD Biosciences, Berkshire UK. The AlexaFluor 488 PNA and propidium iodide dye were purchased from ThermoFisher, UK. The progesterone (P4) and prostaglandin E1 (PGE1) were dissolved in DMSO. The final concentration of the DMSO in an experiment is maximally 0.02% *v*/*v*. The concentration of the compounds was 10 μM P4, 5 mM PGE1, 30 mM ammonium chloride (NH_4_Cl), 10 mM 17α-hydroxyprogesterone (P4OH) and 5 mM prostaglandin E2 (PGE2). The concentrations of the CatSper agonist are at EC_100_ values.

### 4.2. Solutions

The HTF was composed of 72.8 mM NaCl, 4.69 mM KCl, 0.2 mM MgSO_4_, 0.37 mM KH_2_PO_4_, 2.04 mM CaCl_2_, 0.33 mM Na-pyruvate, 12.4 mM Na-lactate, 2.78 mM glucose, 21 mM HEPES, 25 mM NaHCO_3_ and 3 mg/mL BSA, adjusted to pH 7.4 with NaOH, and the osmolarity adjusted to ~275 mOsm using NaCl.

Additionally, the HTF-High K^+^ was prepared by increasing the concentration of the KCl to 30 mM, while the pH and osmolarity were adjusted using NaOH and reducing NaCl by 30 mM, respectively.

### 4.3. Sperm Samples 

Following informed consent, samples for research were obtained from patients undergoing investigation and treatment at the Assisted Conception Unit (ACU), Ninewells Hospital, Dundee, and that were surplus to clinical requirement. Samples from healthy volunteer research donors with normal sperm motility parameters in agreement with World Health Organization 2010 criteria [103] were used in this study under the same ethical approval. All research samples were analysed in line with guidance for human semen studies [104].

### 4.4. Preparation of Donor Samples

Healthy volunteer donors meeting the WHO normal semen criteria (Cooper et al., 2010) adhered to an abstinence period of 2 to 5 days, prior to the sample collection by masturbation in a sterile plastic container. The sample was then allowed to liquefy for 30 min at 37 °C, prepared by density gradient centrifugation (DGC).

For the DGC, 1 mL of liquefied semen was layered on top of percoll gradients (1 mL 40% PureCeption underlaid with 1 mL 80% PureCeption) and centrifuged at 300× *g* for 20 min. The pellet was removed and washed using 5 mL SWM at 300× *g* for 10 min. After washing, the cells were resuspended in human tubal fluid (HTF, CooperSurgical) medium and left to incubate (37 °C, 5% CO_2_) in capacitating conditions for 3 h. For experiments using high concentration of K^+^, the cells were capacitated in normal HTF for 3 h (37 °C, 5% CO_2_), centrifuged and resuspended in HTF K^+,^ with the appropriate agonists, for 1 h.

### 4.5. Preparation of Patient Sperm

The patients’ cells were prepared according to the standard operating procedures employed by the Assisted Conception Unit at Ninewells Hospital, Dundee [30]. The patient sperm cells that were surplus to the requirement for the IVF were collected from the Assisted Conception Unit and resuspended in HTF prior to experimentation.

### 4.6. Computer-Assisted Sperm Analysis

The prepared spermatozoa were incubated for 3 h at 37 °C in capacitating conditions and then mixed with DMSO (vehicle control, 0.02% final concentration), ionophore or P4/PGE1/NH_4_Cl. The motility was assessed using four-chamber, 20-μM deep slides (CellVision, The Netherlands). At least 200 sperm cells were analysed per chamber, per condition. The motility readings were recorded intermittently over 30 min (CASA; CEROS machine [version 12], Hamilton Thorne Research, Beverly, MA, USA). The parameters measured included the progressive motility (PM), total motility (TM), and hyperactivated motility (HA). Proprietary algorithms on the CASA determined the percentage of cells displaying the HA automatically. Specifically, a subpopulation of the sperm displaying a curvilinear velocity (VCL) ≥150 μm·s^−1^, linearity <50%, and an amplitude of lateral head displacement ≥7 μm of algorithms were designated as hyperactive [88].

### 4.7. Determination of Acrosome Reaction by Flow Cytometry

The acrosome reaction experiments were conducted after the sperm were incubated in the capacitating conditions for 3 h. The sperm cells (5 × 10^5^/mL) were then incubated with the agonists (10 μM P4, 5 μM PGE1, 30 mM NH_4_Cl), alone or in combination, in HTF for 60 min (37 °C, 5% CO_2_). The data in Figure 1 are derived from 30- and 60-min incubation times. Subsequently, fluorophore conjugated antibodies to detect the acrosome reaction, either APC-conjugated anti-CD46 (0.05 μg/mL) or PNA-FITC (1 μg/mL), were added and incubated with all the cells for a further 30 min (37 °C, 5% CO_2_). The live/dead stain propidium iodide (PI, 0.8 μg/mL) was added to the cells just before the flow cytometry analysis. A minimum of 10,000 events were recorded using the BD Fortessa LSR II with a blue 488 nm laser (BP 530/30) and a red 640 nm laser (BP 670/14) in the forward-scatter area and side-scatter area. The single cells were gated using the forward-scatter height and forward-scatter area. The live cells were then gated as PI negative, from which the CD46/PNA positive were gated as acrosome-reacted, using the Flow Jo 10. The data are expressed graphically as the percentage of the live acrosome-reacted cells and presented in the Supplementary Table S1 as fold-increase, which was calculated by dividing the induced percentage by the CD46 control percentage.

### 4.8. Intracellular Calcium([Ca^2+^]i) Measurement

In total, 3 × 10^6^ sperm per ml were incubated in the HTF for 3 hrs. The cells were then loaded with the low Ca^2+^-affinity [89] dye Fluo-5N (4.5 mM, Thermo Fisher Scientific, Inchinnan, Renfrew, UK) and left to incubate for 20 min (37 °C, 5% CO_2_) before the centrifugation at 300× *g* for 10 min. The Fluo-5N was selected for these experiments in order to permit the detection of the maximum [Ca^2+^]i response [105]. The supernatant was removed, and the pellet resuspended in 1 mL HTF. A further centrifugation step at 500× *g* for 5 min was performed to ensure the excess dye was removed. The supernatant was removed, and the sperm were resuspended in 1 mL HTF and kept at 37 °C, 5% CO_2_ before experimentation. The 3 × 10^5^ cells were placed into the wells of a 96-well, black bottom plate preheated to 30 °C. The fluorescence measurements of the [Ca^2+^]i were carried out using a FLUOstar Omega reader (BMG Labtech, Offenburg, Germany) at 30 °C. The filters were set to 488 nm (excitation), and 520 nm (emission). The baseline fluorescence was recorded for 30 s prior to the addition of the compounds.

To demonstrate that the P4 and PGE1 responses do not cross-desensitise, the experiments were carried out in accordance with the published protocols [45,106]; the P4 was added after a 30 s recording of the baseline fluorescence, followed 5 min later by the addition of the PGE1 compound, or vice versa. The experiments to demonstrate the CatSper desensitisation involved either the addition of P4, followed by 17α-hydroxyprogesterone (P4OH), or the addition of PGE1, followed by prostaglandin E2 (PGE2). The protocol used to demonstrate the desensitization of both modes of the CatSper activation was similar and involved either the addition of P4/PGE1 followed by P4OH/PGE2, or vice versa. To investigate the effect of asynchronously activating the CatSper with the P4, PGE1 or P4/PGE1 and NH_4_Cl, the agonists (P4, PGE1 or P4/PGE1) were added after a 1-min recording of the baseline fluorescence, followed 5 min later by the addition of NH_4_Cl, or vice versa. In all the experiments, the readings from an additional time-control well (basal fluorescence) were taken, as were readings from a well that was exposed to the agonist(s) at the time point that matched the time point of the addition of the second agonist(s) in the paired experiment.

The normalisation of the background-corrected fluorescence data was, as described previously [90], using ΔF = ((F − Fbl)/Fbl) × 100%, where ΔF is the percentage change in intensity; F is the fluorescence intensity at time t; and Fbl is the mean baseline fluorescence prior to the compound addition. The fluorescence data were normalized to the maximum ΔF response to the ionophore and the data expressed as ΔF (% ionophore). In some of the experiments, the individual curves were analysed using the area under the curve (AUC) function in the GraphPad Prism as a proxy for the total [Ca^2+^]i. The peak [Ca^2+^]i response was defined as the maximum [Ca^2+^]i occurring immediately after the agonist addition.

### 4.9. Intracellular pH (pHi) Measurements

The measurement of the pHi was based on the previous methods [107,108]. After 3 h in the HTF, the aliquots of the sperm cells (4 × 10^−6^ per mL) were incubated with 2 μM 2′,7′-bis(2-carboxyethyl)-5,6-carboxyfluorescein (BCECF-AM) (ThermoFisher, Paisley, UK) for 30 min at 37 °C. The cells were centrifuged for 3 min at 500× *g;* the supernatant was removed; and the cells were then resuspended into the HTF, buffered at pH 7.4. For calibration, five aliquots of the spermatozoa were placed into the HTF and buffered at 6.0, 6.5, 7.0, 7.5 and 8.0, respectively. A FLUOstar Omega plate reader (BMG Labtech) was used to detect the emitted fluorescence (excitation wavelength ratio 490/440 nm and emission wavelength 530 nm, 8 s cycle time, 30 °C). The cell calibration was achieved following the cell lysis by the addition of 1% Triton X-100; a reading was taken from each well; and a calibration curve was constructed. The fluorescence measurements for the control wells (cells + 1% DMSO) and those containing the compounds (10 μM P4, 10 μM and/or 30 mM NH_4_Cl) were recorded using the 490/440 ratios. The unknown pHi values were calculated from the five-point standard curve.

### 4.10. Fertilization Rate at IVF

The oocytes were considered normally fertilized when two pronuclei (2PN) and two distinct or fragmented polar bodies were observed. Following the IVF, the fertilization rate was calculated from the number of oocytes normally fertilized, divided by the total number of inseminated oocytes. The fertilization rate was calculated where four or more mature oocytes (metaphase II) were present.

### 4.11. Statistical Analysis

The data are presented as the mean ± SEM unless stated. Unless indicated, the statistical significance was determined using a one-way ANOVA, followed by Tukey’s post-test, using the statistical package GraphPad Prism 5, and is indicated as * <0.05, ** <0.01, *** <0.001.

## Data Availability

The data underlying this article will be shared on reasonable request to the corresponding author.

## Supplementary Materials

The following supporting information can be downloaded at: https://www.mdpi.com/article/10.3390/ijms231911253/s1.
